# UNICEF UK Baby Friendly Initiative: Providing, receiving and leading infant feeding care in a hospital maternity setting—A critical ethnography

**DOI:** 10.1111/mcn.13114

**Published:** 2021-01-20

**Authors:** Anna Byrom, Gill Thomson, Mark Dooris, Fiona Dykes

**Affiliations:** ^1^ School of Community Health and Midwifery University of Central Lancashire Preston UK; ^2^ Maternal and Infant Nutrition and Nurture Unit (MAINN) University of Central Lancashire Preston UK

**Keywords:** Baby Friendly Hospital Initiative, ethnography, breastfeeding, breastfeeding support, qualitative methods, infant feeding

## Abstract

Although breastfeeding is known to improve health, economic and environmental outcomes, breastfeeding initiation and continuation rates are low in the United Kingdom. The global WHO/UNICEF Baby Friendly Hospital Initiative (BFHI) aims to reverse declining rates of breastfeeding by shifting the culture of infant feeding care provision throughout hospital maternity settings. In the United Kingdom, the global BFHI has been adapted by UNICEF UK reflecting a paradigm shift towards the experiences of women and families using maternity services. This research used a critical ethnographic approach to explore the influence of the national UNICEF UK Baby Friendly Initiative (BFI) standards on the culture of one typical maternity service in England, over a period of 8 weeks, across four phases of data collection between 2011 and 2017. Twenty‐one staff and 26 service users were recruited and engaged in moderate‐level participant observation and/or guided interviews and conversations. Basic, organising and a final global theme emerged through thematic network analysis, describing the influence of the BFI on providing, receiving and leading infant feeding care in a hospital maternity setting. Using Antonovsky's sense of coherence construct, the findings discussed in this paper highlight how the BFI offers ‘informational’ (comprehensible), ‘practical’ (manageable) and ‘emotional’ (meaningful) support for both staff and service users, strengthened by effective, local leadership and a team approach. This is juxtaposed against the tensions and demands of the busy hospital maternity setting. It is recommended that ongoing infant feeding policy, practice and leadership balance relational and rational approaches for positive infant feeding care and experiences to flourish.

**Key messages**

- The revised UNICEF UK Baby‐Friendly Initiative (BFI) standards, alongside effective local leadership and a team approach, offer staff and service users informational, practical and emotional resources to enhance infant feeding care provision and experiences.
- Balancing rational health policies and interventions, such as the Baby‐Friendly Hospital Initiative (BFHI), with relational approaches has the potential to transform organisational cultures.
- Busy postnatal wards in hospital maternity units create challenges for midwives trying to offer optimal infant feeding care to mothers.

## 1 INTRODUCTION

Breastfeeding improves multiple outcomes across health, economic and environmental parameters (Rollins et al., 2016; Victora et al., 2016). The consequences of *not* breastfeeding, for children, range from increases in mortality as a result of infectious diseases (Sankar et al., 2015) to increased hospital admissions for respiratory disease, gastroenteritis (Horta & Victora, 2013) and otitis media (Bowatte et al., 2015). There are also higher rates of childhood diabetes and obesity (Horta, Loret de Mola, & Victora, 2015) and dental disease (Peres, Cascaes, Nascimento, & Victora, 2015; Tham et al., 2015) for children that were not breastfed. Women who do not breastfeed are at an increased risk of breast and ovarian cancer and diabetes (Chowdhury et al., 2015). Despite these consequences, breastfeeding rates, around the world, are slow to increase and, in some areas, continue to decline (Victora et al., 2016; UNICEF/WHO, 2017). Globally, only 41% of infants under 6 months of age are exclusively breastfed (WHO/Unicef, 2019). In the United Kingdom (UK), breastfeeding initiation rates are 74%, dropping to 42% at six to 8 weeks with exclusive breastfeeding rates less than 1%, at 6 months postnatal (NHS, 2019; UNICEF, 2016). Improving breastfeeding rates, through optimal infant feeding care provision, has and continues to be a global and national priority.

In 1989, the World Health Organisation (WHO) and UNICEF published a joint statement: ‘Protecting, Promoting and Supporting Breastfeeding’ (WHO/UNICEF, 1989) detailing a set of best practice standards, referred to as the ‘Ten steps to Successful Breastfeeding’ (Ten Steps). The aim of these ‘Ten steps’ was to reverse declining breastfeeding rates and suboptimal infant feeding care provision by transforming the organisational cultures of hospital maternity settings (WHO, 1990). Based on the ‘Ten steps’, the Baby Friendly Hospital Initiative (BFHI) is promoted as a global health programme that offers a structured mechanism to energise local maternity hospitals to transform their infant and young child feeding practices. A recent systematic review highlighted the benefits of implementing BFHI, establishing the dose–response relationship between the number of BFHI steps women are exposed to and improved breastfeeding outcomes (Pérez‐Escamilla, Martinez, & Segura‐Perez, 2016).

Most recently, an in‐depth review of the global BFHI was undertaken to assess the influence of implementation (UNICEF/WHO, 2017; WHO, 2017a, 2017b). Not only the review confirmed the value of the BFHI for protecting, supporting and promoting breastfeeding, but also it outlined some of the challenges in sustaining high standards of care throughout facilities beyond initial BFI designation (WHO, 2017a). Identified challenges generally related to funding and resource constraints leading to variations in global coverage, internal monitoring and implementation of all the steps (WHO, 2017a). Those steps requiring increased staff training, audit and assessment were being more difficult to implement and sustain due to time and resource restraints (WHO, 2017a). This work led to the global BFHI ‘Ten steps’ being revised and in places reworded to reflect the best available evidence (WHO, 2018). The changes are summarised by Aryeetey & Dykes, 2018, see Table 1).

**TABLE 1:** WHO/UNICEF ‘Ten steps’ to successful breastfeeding (original 1989 versus revised version 2018)—adapted from Aryeetey and Dykes (2018)

Step	Original version (1989)	Revised version (2018)
	‘Every facility providing maternity services and care for newborn infants should’:	
1	Have a written breastfeeding policy that is routinely communicated to all health care staff.	(a) Comply fully with the International Code of Marketing of Breast‐milk substitutes and relevant World Health Assembly resolutions.
		(b) Have a written infant feeding policy that is routinely communicated to staff and parents.
		(c) Establish ongoing monitoring and data‐management systems.
2	Train all health care staff in the skills necessary to implement the breastfeeding policy.	Ensure that staff have sufficient knowledge, competence and skills to support breastfeeding
3	Inform all pregnant women about the benefits and management of breastfeeding.	Discuss the importance and management of breastfeeding with pregnant women and their families
4	Help mothers to initiate breastfeeding within half an hour of birth.	Facilitate immediate and uninterrupted skin‐to‐skin contact and support mothers to initiate breastfeeding as soon as possible after birth.
5	Show mothers how to breastfeed and how to maintain lactation even if they are separated from their infants	Support mothers to initiate and maintain breastfeeding and manage common difficulties.
6	Give newborn infants no food or drink other than breastmilk, unless medically indicated.	Do not provide breastfed newborn infants any food or fluids other than breastmilk, unless medically indicated
7.	Practice rooming‐in, allowing mothers and infants to remain together 24 h a day.	Enable mothers and infants to remain together and to practice rooming‐in 24 h a day.
8.	Encourage breastfeeding on demand	Support mothers to recognise and respond to their infant's cues for feeding.
9.	Give no artificial teats or pacifiers (also called dummies or soothers) to breastfeeding infants.	Counsel mothers on the use and risks of feeding bottles, teats and pacifiers.
10.	Foster the establishment of breastfeeding support groups and refer mothers to them on discharge from the hospital or clinic.	Coordinate discharge so that parents and their infants have timely access to ongoing support and care.

Although there are over 15,000 BFHI designations across 152 UN member states, coverage within most countries has remained low (UNICEF, 2016; WHO, 2018). There have been moves to understand the barriers and facilitators to implementing the BFHI policy, as captured in two reviews of the research evidence (Schmeid et al., 2014; Semenic, Childerhose, Lauzière, & Groleau, 2012) and research exploring how BFHI training influences staff attitudes and behaviours (Dagvadorj, Yourkavitch, & Lopes, 2017; Martens, 2000; Owoaje, Oyemade, & Kolude, 2002). Despite these reviews, there has been limited exploration of the influences of the BFHI in the UK context.

The BFHI was implemented within the UK in 1994, by UNICEF UK and was renamed the Baby Friendly Initiative (BFI) to emphasise the extended scope beyond the hospital setting (Unicef UK, 2013). A recent mixed‐methods systematic review examined the impact of the national BFI implementation (hospital and community) on maternal and infant health outcomes, in the UK, concluding that the UNICEF UK BFI increases breastfeeding rates up to 6 weeks (Fallon, Harrold, & Chisholm, 2019). The review also noted the importance of the global BFHI being ‘situationally modified in resource rich settings’, that is, adapted to respond to local contextual issues such as the long‐standing bottle‐feeding culture influencing infant feeding practices in the UK (Brown, 2015, 2016). This review identified several research gaps including a need to explore how implementing the BFHI standards influences the organisational cultures in hospital settings.

In 2012, UNICEF UK BFI reviewed and revised their BFI standards (see Table 2). This was in response to evidence highlighting the importance of *how* women and families' experience infant feeding care (Unicef UK, 2013).

**TABLE 2:** UNICEF UK revised BFI standards (2012)

Maternity	Neonatal	Health visiting	Children centres
1. Support pregnant women to recognise the importance of breastfeeding and early relationships for the health and well‐being of their baby	1. Support parents to have a close and loving relationship with their baby	1. Support pregnant women to recognise the importance of breastfeeding and early relationships for the health and well‐being of their baby	1. Support pregnant women to recognise the importance of breastfeeding and early relationships for the health and well‐being of their baby
2. Support all mothers and babies to initiate a close and loving relationship and feeding soon after birth	2. Enable babies to receive breastmilk and to breastfeed when possible	2. Enable mothers to continue to breastfeed for as long as they wish	2. Protect and support breastfeeding in all areas of the service
3. Enable mothers to get breastfeeding off to a good start	3. Value parents as partners in care	3. Support mothers to make informed decisions regarding the introduction of food or fluids other than breastmilk	3. Support parents to have a close and loving relationship with their baby
4. Support mothers to make informed decisions regarding the introduction of food or fluids other than breastmilk		4. Support parents to have a close and loving relationship with their baby	
5. Support parents to have a close and loving relationship with their baby			

The 2012 revised UNICEF UK BFI standards represent a paradigm shift with a purpose to support the implementation of evidence based, mother‐centred best practice standards in health care settings, designed to support all families with infant feeding and relationship building. The aim of these revised standards is to create a mother–baby and family‐friendly culture of infant feeding care provision to improve care practices and experiences (Unicef UK, 2013, 2015).

There is growing interest, throughout the UK, in changing organisational culture as a lever for health care improvement (Davies & Mannion, 2013; Tate, Donaldson‐Feilder, Teoh, Hug, & Everest, 2014). Organisational culture has been defined from a range of perspectives (Frith, Vehvilainen‐Julkunen, Beeckman, Loytved, & Luyben, 2014). Most definitions focus on the shared attributes between the members of a group or as Davies (1984, p. 1) describes ‘a pattern of shared beliefs and values that gives members of an institution meaning, and provides them with the rules for behaviour in their organisation’. Yet what is explicitly shared on the surface may not reflect the variance within groups restricting nuance and deeper understanding. Mannion and Davies (2018, p. 2) argue that ‘healthcare organisational culture’ is a metaphor for some of the softer, less visible, aspects of health service organisations and how these become manifest in patterns of care’. Exploring the visible and more subtle ways that BFI policy influences practice will offer insight into how large‐scale health interventions such as the BFI can be used to levy cultural change.

Culture has been identified as both the ‘culprit’ and ‘remedy’ for health care challenges (Mannion & Davies, 2016, 2018). The BFI is promoted as a ‘remedy’ for improving infant feeding culture throughout hospital maternity services to enhance breastfeeding rates. Understanding how interventions, such as the BFI, influence the organisational cultures of hospital maternity settings can help to inform the development of appropriate policies and practice to transform staff and service–user experiences.

There are currently no studies that have explored the influence of these revised national BFI standards on the organisational culture of maternity services. Including how the national BFI influences the beliefs, practices, perceptions and experiences of women, families and staff who provide or receive infant feeding care in BFI accredited hospital maternity services in the UK. Rather than focusing on *how* the national BFI is implemented, we explored the influence the standards appeared to have on the organisational cultures of one hospital maternity setting, primarily in the postnatal ward environments.

### 1.1 Study aims and objectives

The aim of this study was to explore how the BFI influenced the organisational cultures of one maternity unit in the North of England. The specific objectives of the study were to examine whether and in what ways the BFI influenced the beliefs, practices, views and tacit assumptions of the maternity staff and explore the perceptions and experiences of service users being cared for in the maternity services and how the changes to the BFI standards and policy influence care practices.

## 2 METHODS

### 2.1 Theoretical perspectives

A critical theory lens was used to inform the study design and approach. Critical theory enables social scientists to examine beneath the appearance of given social positions towards new social commentaries and understanding (Kellner, 1989) and therefore has value in supporting new understandings of how the revised BFI, in the UK, influences the culture of infant feeding care in hospital maternity settings. Critical theory researchers see all interactions and disciplines as manifestations of power relations linked to the social and historical contexts that produced them (Crossley, 2005). BFI has a long history as a global intervention that aims to ‘protect, promote and support’ breastfeeding; indeed, the study site included had engaged in the BFI since its inception in the UK and had its own social and historical context for consideration. Adopting a critical theory perspective helped inform a detailed exploration of the BFI within this local context including how it influenced infant feeding practice throughout the busy environment of a hospital maternity service, over time. As in other research aiming to explore cultures in maternity care provision (Dykes, 2009a), critical theory influenced the selection of the methodology and tools selected to gather, analyse and report the data.

### 2.2 Conducting the ethnography—Participating in the ‘fast‐food service’

Critical ethnography is a methodology that is suitably aligned to the theoretical perspective of critical theory (Thomas, 1993). A critical ethnographic approach was employed in order to explore how the BFI influenced the organisational culture of infant feeding in a hospital maternity setting. The concept of culture developed by critical ethnographers generally describes culture as a complex creation of activities such as routines, rituals and actions (Frith, Vehvilainen‐Julkunen, Beeckman, Loytved, & Luyben, 2014). In relation to the BFI and infant feeding care, these might include the daily infant feeding actions and activities that collectively can be considered a series of routines or rituals, forming part of everyday practices and experiences. Critical ethnography encourages consideration of the individual level cultural concepts alongside broader organisational and societal perspectives. As such, it offered an ideal approach to probing micro (individual feeding tasks or behaviours) to macro (organisational feeding policies and processes)‐level issues relating to the influence of the BFI on the organisational culture of a hospital maternity unit.

Specifically, critical ethnography was used to elicit various levels of cultural knowledge both explicit (easily seen), such as infant feeding interactions, and tacit (hidden) such as people's feelings and perceptions, as recommended by Spradley (1980). The aim was to explore the ‘visible manifestations (artefacts)’ of maternity care culture, alongside the ‘shared ways of thinking’ about the BFI and associated infant feeding care including the values and beliefs that underpin actions and behaviours, while also examining the ‘deeper shared assumptions’ which mark the unconscious and unexamined aspects of everyday practice (Mannion & Davies, 2016, 2018). Ethnographic methodology enabled direct observation of the cultural setting and ways in which BFI is implemented and experienced by staff and service users.

### 2.3 Ethical considerations

Full ethical approval was granted through the lead authors faculty of health ethics committee (proposal 186) and via the national research ethics service (REC reference: 11/NW/0202). Management approval was also granted by the participating hospital research and development unit in accordance with NHS research governance arrangements. Ethical issues in relation to informed consent, confidentiality, data protection and withdrawal were adhered to throughout this study.

### 2.4 Study site, participants and recruitment

The study site selected is described as a large maternity unit (over 6000 births per year) offering consultant‐led, birth centre and specialist services, such as a transitional care unit. The decision was made to conduct the study at a single site, over a number of years, to enable a review of how the revised Unicef UK BFI policy, introduced in the UK during the study duration, influenced how infant feeding care was provided and received. This offered a unique opportunity to explore the experiences and perceptions of staff and service users, who were offering or receiving care in a maternity service engaged with these revised BFI standards.

The study site had sustained BFI accreditation for 20 years, achieving the BFI sustainability gold award towards then end of the study. Between 2011 until 2017, breastfeeding initiation rates increased by 5% to 79% overall. Women with uncomplicated pregnancies and births were generally cared for in the midwifery‐led birth centres and discharged directly home. As such, the postnatal ward was populated with mothers and babies with complex health or social care needs. The maternity unit served a predominantly White population with some women of South Asian origin. The communities accessing the service came from disparate socio‐economic backgrounds.

Following heads of service approval, maternity staff were approached to establish their willingness to be included in the study. Maternity staff were included if they were expected to work with the BFI standards in their daily practice and consented to inclusion. Service users were approached in the antenatal and postnatal areas of the service, following identification for suitability with staff. Service users and their families were included if they could speak English, were being cared for on the postnatal ward and had consented to inclusion. There were no specific exclusion criteria or sample size. The focus was to elicit a broad range of perspectives and experiences. A convenience sample of 26 maternity staff (*n* = 16 midwives, *n* = 2 maternity care assistants, *n* = 8 infant feeding team members) and 21 service users (*n* = 16 mothers, *n* = 5 fathers) consented and participated in the study. This number reflected those staff and service users available and consented for participation during the study phases described below. Table 3 offered details of the participant characteristics.

**TABLE 3:** Study participant characteristics

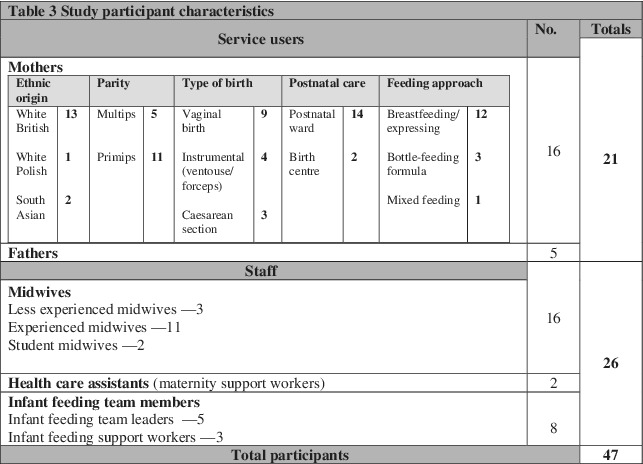

### 2.5 Data collection

Data were gathered through moderate‐level participant observations of infant feeding activities and care provision predominantly in the postnatal areas of the hospital maternity unit, over a total period of 8 weeks, split over four phases of time between 2011 and 2017. Due to funding and time restrictions, data were gathered in 2‐week blocks, over these phases. Gathering data in these shorter 2‐week bursts, aligns with emerging rapid ethnography approaches that are being used to capture the complexities of service provision, the implementation of new health care technologies and programmes, including the nuanced practices of care provision, in more limited time frames (Ackerman et al., 2017; Mullaney, Pettersson, Nyholm, & Stolterman, 2012). Although this posed some challenges, in terms of the prolonged exposure recommended by traditional approaches to ethnography, the size and busyness of the maternity unit did enable access to a variety of participants and BFI practice. Care was taken to observe care across all shift patterns to help increase exposure to a range of infant feeding practices. The 2‐year gap between each data collection phase was required in order to understand how implementation of the revised BFI standards influenced practice.

As this was a doctoral research project, the participant observations were conducted by the lead author acknowledging that her role as participant observer was at once both insider (as a previous midwife, infant feeding lead and current midwifery educator) and outsider as a novice researcher. This involved exploring what people said alongside what people did (Spradley, 1980). Moderate‐level participation encouraged a balance between observing and participating, not only focusing on observing care as an onlooker but also responding and answering simple questions or assisting service‐users by asking for extra support, if required (Spradley, 1980).

Moderate‐level participant observation was conducted in key clinical maternity environments where the BFI steps were expected to be performed. This included the birthing spaces, antenatal area and postnatal wards. Some of the meetings and training related to the BFI were also attended and observed. Most of the observations were conducted on the postnatal ward, where majority of the maternity BFI standards are implemented. Observations were made from a macro perspective (sitting in general areas such as communal workspaces and corridors) and then the micro perspective (in the bays, rooms and at the bedside of service users during periods of care interactions). Focus was placed on interactions between ward staff, mothers, babies and families, especially during moments of infant feeding care provision. Care was taken to undertake participant observations during day and night shifts over the four phases of data collection. When engaging in the participant observation fieldwork, Spradley's (1980, p. 78) nine dimensions of social situations (Table 4) were used to inform the focus of the observations and to help guide initial analysis.

**TABLE 4:** Spradley's (1980) nine dimensions of social situations

Dimension	Description
1. Space	The physical place or places—for example, looking at the ward environment, the bed space, the clinical areas and hand‐over room.
2. Actor	The people involved—for example, all the consenting staff, women and family members on the postnatal ward area.
3. Activity	A set of related acts people do—for example, the daily routines of ward life, that is, admission to the ward support, the daily postnatal checks performed and routine infant feeding support.
4. Object	The physical things that are present—for example, the resources available for supporting feeding (leaflets, doll and breast models and express pumps).
5. Act	Single actions that people do—for example, the expressions that people make and the movement's people make (i.e., supporting breastfeeding, adjusting baby's position during infant feeding and taking babies out of the room for medical checks)
6. Event	A set of related activities that people carry out—for example, the handover each day on the wards and the shift as a whole.
7. Time	The sequencing that takes place over time—for example, how much time is spent supporting infant feeding or in other ward activities.
8. Goal	The things people are trying to accomplish—for example, the specific goals ward staff might have.
9. Feeling	The emotions felt and expressed—for example, how do the parents or staff feel throughout the specific shifts observed.

Spradley's dimensions were used to guide the development of field notes throughout all periods of observation, especially the initial phases of data collection, during which the aim was to understand the context and setting for the BFI standards and infant feeding care provision. Initial broad observations were followed with a series of focused observations and interviews: used to explore dialogue and care provision from the micro perspective. They helped to elicit information pertaining to the narratives involved in service user‐to‐staff interactions and vice versa relating to the BFI standards and associated infant feeding care provision.

Where possible, these observations were recorded in field notes and/or via a Dictaphone to ensure both the narrative and nonverbal behaviours were captured. This enabled greater accuracy of translation. These observations were sustained for as long as the period of care being provided lasted and, for the most part, happened by the mother and baby's bedside. Occasionally, they occurred from a location in the communal ‘bays’ where a few women and babies received their care.

Alongside these observations, short interviews were conducted, as appropriate and necessary, to help clarify participant actions, thoughts and feelings related to particular care experiences. Such interviews helped to check understanding and clarify researcher inferences made during the observations. Hammersley and Atkinson (2007) argue that in ethnography, observations and interviewing are essential and mutually beneficial enabling an iterative process where one informs the other and vice versa. As such, the general and specific questions asked, during these short interviews, helped to deepen understanding of the interactions observed. These interviews were recorded either electronically or via my field notes as appropriate.

In addition to gathering data via observations and interviews, relevant documentation, related to the BFI policy, guidelines and wider infant feeding care, were reviewed. The BFI encourages maternity units to adopt specific policies, paperwork, checklists and record keeping strategies. As such, it was valuable to look at these in combination with the observations and interview data gathered. These documents supplemented what was observed and heard, adding another layer to the data collection and broader appreciation of the influence of the BFI in practice.

### 2.6 Data analysis

Data collection and analysis occurred concurrently, in an iterative process. Initially, data were transcribed and uploaded into MAXQDA data management software. Using MAXQDA, each transcript was read, line‐by‐line, attributing initial codes and labels that helped to transform the data into manageable and meaningful coding framework. From these initial codes, inductive, thematic analysis was used to generate basic, organising and global thematic networks, as described by Attride‐Stirling (2001). Thematic networks are underpinned by Toulmin's (1958) argument theory ensuring the establishment of ‘claims’ (global theme) based upon clear ‘warrants’ (organising themes) with established ‘backing’ (basic themes). Utilising thematic networks offered a practical and structured approach to generating, confirming and communicating the findings. The process of identifying basic themes and then synthesising these in to organising themes helped to shape a global perspective regarding how the BFI appeared to influence the culture of infant feeding care within the hospital maternity unit studied. Through reflexivity, care was taken to avoid the temptation to overgeneralise, overtheorise or oversimplify the data collected.

### 2.7 Reflexivity

Reflexivity involves recognising that the researcher is influenced by their socio‐cultural background and personal values and beliefs (Freire, 1972). Reflexivity was addressed by AB maintaining a reflexive diary through audio recording reflections throughout the whole process of the research. Supervisory meetings were used to explore this reflexive positioning with FD, GT and MD encouraging deeper interrogation of perspectives, feelings and experiences. Consideration of previous BFI and infant feeding experiences including the bias these could create was outlined and discussed.

### 2.8 Ethical considerations

Full ethical approval was granted through the lead authors faculty of health ethics committee (proposal 186) and via the national research ethics service (REC reference: 11/NW/0202). Management approval was also granted by the hospital trust research and development unit in accordance with NHS research governance arrangements. Ethical issues in relation to informed consent, confidentiality, data protection and withdrawal were adhered to throughout this study.

## 3 RESULTS

**FIGURE 1:**
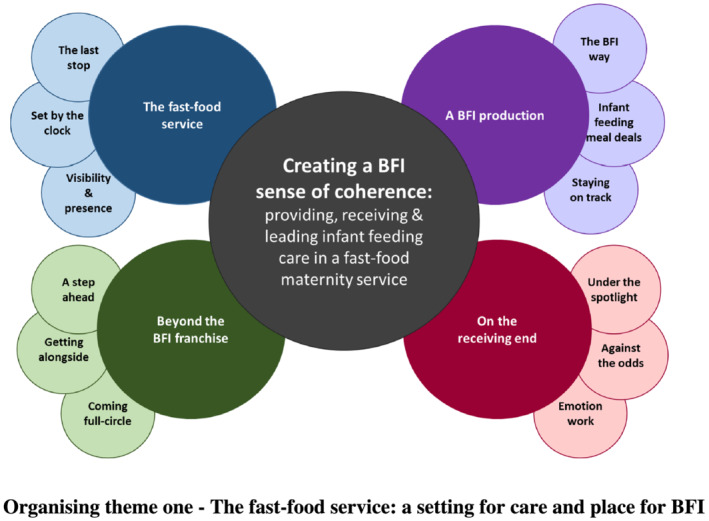
Thematic network of global, organising and basic themes

Following Attride‐Stirling's (2001) thematic network approach to data analysis, four organising themes developed from a range of basic themes as captured in Figure 1. Data presented in this paper have been selected to reflect the diversity and range of data gathered across the study.

### 3.1 Organising theme one—The fast‐food service: A setting for care and place for BFI

This organising theme highlights the general activity on the postnatal ward and how infant feeding appeared and was considered within a fast‐pace, service delivery environment of the maternity hospital setting. The postnatal ward was considered by some participants to be ‘a strange place to work in … and really busy … a fast pace’ (Julie, Infant feeding lead, phase 1); the evidence and influence of which was described through three basic themes (see Figure 1).

#### 3.1.1 The last stop on a medical conveyor‐belt

The postnatal ward appeared as the last stop on a medical conveyor‐belt of maternity care provision. The increased medical needs of a ‘complex crowd’ of service users seemed to influence the approach to care provision and how the BFI standards were performed and received in the hospital maternity setting. This was captured during an interview with Julie:

this is a busy ward isn't it you've got, you've got high risk? There's something going on with all these mothers and babies … it's really busy, really noticeable now you've stripped back kind of normality (Julie, Infant feeding lead, phase 1).

During a night‐shift observation, Judith, a senior midwife, was trying to manage women with complex health needs and referred to the postnatal ward as being ‘the bottom of the pecking order' in relation to other areas of the maternity services. Staff referenced the 'conveyor belt' and factory‐organised care of the ward environment, the high‐risk status of the mothers and babies and pressure to process service users through the system:

We need beds, we need bed, you need to get people out. Why are you not discharging people? … … Conveyor belt. Felt like a factory [referring to the shift]. (Joanne, senior midwife, phase 2).

The service‐driven imperative to process women and babies through the system resulted in staff having to utilise a range of crowd‐control techniques to optimise resources and care delivery.

#### 3.1.2 Set by the clock

The service demands and routines observed were revealed through concepts of time and how it influenced the more general activities on the postnatal ward. The clock was noted to have a physical and metaphorical presence:

The clock on the wall of the ward, hangs directly opposite the midwives' station, a timely reminder for staff to stay on track. The continual beat of the clock hands, marking out the steps towards the next task, the next job, the next thing on the checklist (Field notes, phase 2).

A focus on time and tasks was observed frequently, often experienced as demanding and pressured for both staff and service users. Lucy, a midwife, remarked during a period of observation: ‘the hurry is all the things you have to do six times’, referring to the repetition of tasks and routines against the clock.

Frequently, the varying, complex and unpredictable needs of mothers and babies fell ‘out of sync’ with the clockwork structure of these routines. To process and manage the workload saw staff prioritising ‘caring for the records.’ Record keeping was a persistent activity for all staff, in the postnatal ward environment and appeared to dominate staff time during a shift:

Midwives stood or sat at the midwives' station recording and organising records. This appeared to be ritualistic and consumed a significant amount of time during the period of observation. Frequent reference to paperwork—asking mothers and each other: ‘have you got your paperwork’, ‘have you got your red book’ piles of records strewn over the midwives' station. (Field notes, phase 1).

Staff tended to find the volume of paperwork and ward routines as a barrier to effective infant feeding care and support. Interestingly, staff who prioritised care labelled themselves as ‘deviant’, as captured by Judith, during a period of observation, she said (while writing in the records, after she had spent time caring): ‘I'm naughty because I can spend too much time caring and don't write anything in my notes’ (Judith, senior midwife, phase 2).

The postnatal ward was organised along a series of connected corridors. This, coupled with the busyness of routine medical tasks and activities, influenced the visibility and presence of both staff and infant feeding, generally.

#### 3.1.3 Visibility and presence

This basic theme presents how the specific layout and design of hospital spaces influenced both staff and service user experiences, perceptions, actions and behaviours. There appeared to be ‘variable spaces for care’ and these influenced service user experiences, as described by Gemma, a breastfeeding mother who was cared for in both the private and communal spaces of the postnatal ward:

I'm in a (side) room it feels very different …. I don't feel like I'm shutting myself off from everybody else … That's what it felt like in the dorm [4 bed shared bay] …. it's not nice, you know, you're forever feeling ‘oh, who's going to pop their head round the curtain’. (Gemma, BF mother, phase 1).

The space and place for infant feeding influenced ‘feeding visibility’. The busyness of the postnatal ward seemed to encourage women to close their curtains and avoid breastfeeding during visiting times, also reflecting the pressures of bottle‐feeding culture of local communities in the study site locality and across the UK. This was captured in my reflective log:

It has been surprising that I have yet to see a woman breastfeeding her baby on the postnatal ward unless I'm invited behind her curtains. It is completely invisible apart from a range of posters on the walls' (Reflective log, phase 1).

The pressures of time and ward routines influenced ‘staff visibility and presence’ resulting in a fluctuating physical and temporal absence of staff, especially in the communal areas of the postnatal ward:

A father approaches the midwives' desk with caution—I see him trying to catch someone's attention. One midwife has her back to the ‘corridor’ filing notes. The other midwife sits at the desk, head down, writing in records … neither look up. The man waits, without interrupting. (Field notes, phase 1).

Collectively, ward activities appeared to echo those of a fast‐food service. Staff processed women, babies and visitors through what appeared to be like a ‘drive‐through’ conveyor‐belt environment, delivering packages of information and care along the way, conducted in the busy postnatal ward environment. The ‘fast‐food’, rationalisedsetting influenced how the BFI was sustained on a day‐to‐day basis, issues captured in the next organising theme.

### 3.2 Organising theme two—A BFI production

In this organising theme, issues relating to the BFI production, the everyday care provision associated with the BFI in the fast‐paced hospital environments emerged. This theme captures how, inspite of the busy environment, the BFI standards appeared to have been adopted, influencing the culture of infant feeding practice within the hospital maternity setting. This theme arose from three basic themes, now presented.

#### 3.2.1 The BFI way

The BFI standards appeared to have influenced changes in infant feeding practice. For most staff, involved in the study, there was a clear sense that ‘the BFI way’ was a part of everyday life and practice. This was reported by Jackie:

It's on my mind all the time. It's ingrained and it's part of my work. Every single day wherever I'm working. (Jackie, senior midwife, phase 2).

Midwives appeared to have a ‘natural’ attitude towards promoting breastfeeding and breast‐milk feeding influenced by the BFI standards. One midwife mentioned how the BFI ‘*certainly changed our practice … we do it now without thinking* (Jessica, midwife, phase 1). This reflected the embeddedness of the BFI standards in the maternity unit.

Staff seemed to value the BFI with its logical, rationalised standards. This was outlined by Jackie:

First of all you've got your guidelines with it … the fact it is steps, and, yeah, one step leads to another and then you progress. (Jackie, senior midwife, phase 2).

The BFI standards appeared as a ‘BFI script’, a set of feeding directives, helping to improve breastfeeding outcomes, as described by Janet:

The advice that has been brought in because of the baby friendly initiative, I feel personally over the years has helped me in achieving a more successful outcome of breastfeeding. (Janet, midwife, phase 1).

Occasionally staff and service users were observed to be ‘going off script’ in response to service‐demands, individual care priorities or because they were unfamiliar with the changing BFI script, following introduction of the revised standards in 2012. Fluctuations in practice alignment with ‘the BFI way’ were captured in the next basic theme.

#### 3.2.2 Infant feeding ‘meal deals’

Observations and conversations with staff revealed the various restrictions on ‘feeding time’ and how breastfeeding support arose as a variable pressure on staff time and resources. This created a paradox where staff supported and promoted the values of the BFI yet also found it difficult to support breastfeeding the way they wanted, all of the time, as captured by Anne:

It's not that you don't want to help somebody, you just know the time it's (breastfeeding) is going to take and you haven't got the time (Anne, midwife, phase 1).

Conversely, bottle feeding was generally perceived by staff and service users as being less time‐consuming than breastfeeding. As such, there was the sense that bottle feeding mothers were left to ‘get on with it’ as outlined by one of the midwives:

It's easier for a mum, if she's unsure to say ‘just give a bottle’ … you spend less time with a formula feeding mum because they just get on with it (Shelley, midwife, phase 1).

With the introduction of the new BFI standards in 2012, there appeared to be a shift to offering support to all women and families, regardless of feeding choice, as discussed by Karen, during her interview:

It is my role to ensure all women and babies are offered support to develop close and loving relationships, this includes bottle feeding families. I always ensure bottle feeding mothers get a discharge conversation so they feel informed about responsive bottle feeding and keeping the baby close. (Karen, Infant feeding support worker, phase 4).

Some staff, especially midwives, shared frustration of having to offer ‘bite‐sized packages of support’ due to pressures of workload and restrictions of time:

Judith (midwife) shared how, how frustrating it can feel to be pulled and be unable to get to women that need review of and support with their feeding. And having to do it (support feeding) in five minutes, ten minutes, pieces and bite‐sized pieces of support (Reflective log, phase 2).

Importantly, the women and families, included in the study, regularly reported how staff had ‘time to care’ for them in terms of their infant feeding support needs. This denoted an important difference between the experiences of working or being cared for in this maternity service. Having ‘time to care’ appeared to be easier in the midwifery‐led settings of the maternity service as described by Shawn,

I didn't feel rushed ever she, you know … she were really nice and asked me about other things as well that were going on in my life which were nice (Shawn, BF mother, phase 3).

The general pressures of time and high workloads resulted in staff adopting strategies to ensure they were ‘staying on track—managing and directing the performance’ of BFI.

#### 3.2.3 Staying on track managing and directing the performance

In this basic theme, the management strategies staff employed to ensure BFI standards were maintained in practice arose. Staff appeared hands‐on, during busy times ‘doing’ infant feeding support rather than ‘being‐with’ a mother and baby during infant feeding experiences as captured in my field notes, during a busy shift:

The task‐orientated nature of the workload results in staff going in to rooms to ‘do’ something then returning to their base, at the midwives' station. Breastfeeding support also seems to be treated as a task—one midwife mentioned she was ‘going to “do” breastfeeding now’ (Field notes, phase 1).

With the introduction of the revised standard, there appeared to be a shift in approach to infant feeding care, with a more conversational style of support observed and described. This is explored further in the final organising theme ‘Beyond the BFI franchise’. During busy times or to navigate complex infant feeding challenges, staff were observed ‘passing the book—delegating care’ by distributing leaflets or referring women to the infant feeding volunteers or leads. This was described by Joanne:

Sometimes we have volunteers that appear. But, I mean we use and abuse them all every day you know (laughs). But they're… it's just, in a way that becomes just taking a task off us. (Joanne, midwife, phase 2).

This delegation appeared as essential to manage the high workload demands, while maintaining the BFI standards.

The benefit of working in a BFI accredited unit meant that all members of the health care team receive training to support effective feeding, as articulated by Judith:

I think the good thing is as well, we've got, everybody is trained in it now. Even if the midwife is focussed on an unwell mum or an unwell baby. There are, your Healthcare Assistants. You've got your Support Workers, you've got your Volunteers. (Judith, midwife, phase 1).

To help staff stay on track, the infant feeding leads developed strategies and a series of disseminated support from BFI champions. Directing staff through appropriate training and support was a key aspect of the infant feeding leadership role as highlighted by Julie, the infant feeding lead, during a champions meeting:

Urm regarding that action there regarding birth suite staff what we've done there is developed a little programme for the new band 5 midwives working on birth suite. (Feeding champion meeting, Julie, infant feeding lead, phase 1).

The performance was also directed through regular updates, posters and memos to staff. These helped to motivate staff but also created a BFI pressure to perform, issues captured in the next organising theme.

To maintain ‘the BFI way’, it seemed to be important that staff have comprehensible and manageable steps to follow. The BFI standards offered that. Equally, direction and leadership seemed to help the service stay on track. Overall, the BFI standards did appear to be embedded in daily practice, they had an impact on both staff and service users, as captured in the next organising theme.

### 3.3 Organising theme three—On the receiving end: Infant feeding in a BFI franchise

This organising theme captured the experiences of staff and service users and the ways in which they experienced being on the receiving end of the shifting BFI standards within the ‘fast‐food’ maternity service. These BFI and associated infant feeding practices appeared to impose varying pressures for both staff and service users, presented across three basic themes:

#### 3.3.1 Under the spotlight

Staff and service users experienced a ‘pressure to perform’ the BFI standards alongside other needs and duties. These performance pressures were influenced by the range of surveillance measures adopted to maintain and strengthen the BFI standards in practice. Surveillance, both internal and external, is a significant feature of the BFI. Audits constituted the primary method of surveillance and were referred to frequently by staff participants, who identified them as a source of pressure:

Every member of staff who attends that [BFI] training then has a post‐training audit. So, I then also chase people up to do their audits, which is often a bit of challenge in a busy unit. […] sometimes you can get lost, you can lose sight, when you are in this job and you do your audits and you think ‘Oh god, we're not passing’. (Judith, infant feeding midwife, phase 3).

Despite the pressure, staff also reported a sense of pride from receiving external validation through accreditation, as reflected in an interview with an experienced infant feeding lead, following a recent GOLD sustainability standards accreditation:

The external BFI assessments drive improvements and offer maternity services something to feel proud of beyond being flogged for performance and finances. It keeps maternity services visible at a Trust level and ensures continued resources for infant feeding services (Nicola, BFI senior team member, phase 4).

From these perspectives, the BFI and associated surveillance, creates a positive pressure to sustain BFI standards and maternity service resources.

Surveillance also extended to the observation and monitoring of infant feeding episodes between women and their babies. Women and families appeared to be caught at times between two opposing cultures: the BFI culture of the maternity services and the predominantly bottle‐feeding culture of the wider community. This led to some women changing their behaviours, avoiding breastfeeding during visiting hours. Women also felt a pressure to produce breastmilk especially when required to breastfeed and express to increase the milk supply for sick or vulnerable babies:

It's hard I felt like a bit of a milking cow because I'm feed, feeding and then an hour and a half later I'm expressing, then an hour and a half later I'm feeding. (Adele, BF mother, phase 1).

Yet breastfeeding seemed to continue against the odds of these pressures, as captured in the next basic theme.

#### 3.3.2 Against the odds

The pressures to perform, feed and produce resulted in women and staff appearing to breastfeed ‘against the odds’. This was influenced, in part, by how receptive staff and service users were to the BFI standards and recommendations due to past infant feeding experiences and general institutional and environmental factors. Individual feeding perspectives arose from personal experiences, attitudes and beliefs. Some women shared an ambivalence to infant feeding:

I did think if it really hurts, I won't. I wasn't too fussed, I thought if it really hurts for a week then I wouldn't do it, but then … I would prefer to. (Angela, Mixed Feeding, Phase 2).

For staff, their feeding perspectives were more likely to be influenced by their working context. Over time, staff appeared to be more receptive or accepting of the BFI ensuring that regardless of perspectives, the BFI work was done and ‘the BFI way’ achieved most of the time. Yet the infant feeding leaders, staff and service users were seen to navigate a series of barriers to ensure breastfeeding was supported against the odds. For staff the BFI appeared to save the day offering practical information, helping them to overcome infant feeding challenges:

Work at it and you do it and then before you know it you've accomplished it and you think well, and it saved the day. So, and, and you've sort of … climbed that hill really. (Janet, Senior Midwife, Phase 1).

This staff support and perseverance also seemed to help women to breastfeed against the odds:

They've been really good. Especially with the feeding, ‘cos I struggled to feed her at first, she wasn't feeding, but…yeah, they've been really helpful’ (Angela, BF mother, phase 2).

For leaders, they found they had to overcome resource barriers:

Ok, so we've been thinking what can we do with what we've got, we've no more funding, no more time, so we just have to think differently, just to see … we don't know if it's gonna work but you have to try different things. (Julie, infant feeding lead, phase 1).

Striving to maintain BFI standards and breastfeed against all odds appeared at times to have emotional consequences for staff and service users.

#### 3.3.3 Emotion work

The emotional consequences of working and being cared for in a BFI accredited fast‐food service saw staff experiencing ‘feeling torn’ between ‘being’ a midwife or becoming a ‘medical‐doer’. Being pulled away from supporting breastfeeding had negative consequences for staff, especially midwives, as highlighted by Joanne:

Whatever you're doing you're torn: you have to choose what you do – medical tasks or breastfeeding; it's one or the other (Joanne, midwife, phase 2).

Throughout the study women too, reported ‘feeding highs and lows’ in relation to the emotion work of feeding. This was referenced by Sally, a breastfeeding mother:

It were a bit of an emotional rollercoaster to start with and when they came and said she's lost, like, 12.7 percent of her body weight I were, I was just mortified. I couldn't stop crying all afternoon. (Sally, BF mother, phase 2).

Yet conversely, some aspects of feeding especially skin contact, felt really good for women and families:

It [skin contact] were brilliant, it were brilliant. It didn't last very long, I think probably about five minutes or so but yeah, it were really nice, yeah. (Angela, Mixed Feeding, Phase 2).

This emotion work linked directly to how receptive staff and service users appeared to be to the BFI standards and infant feeding care directives.Caring for staff and service users appeared to be crucial to ensuring high standards of care were achieved. The next organising theme considers how the revised BFI helped to support staff and service users to move ‘beyond the BFI franchise: caring, leading and transforming’.

### 3.4 Organising theme 4—Beyond the BFI franchise: Caring, leading and transforming

In this organising theme, the influence of the new UK BFI standards emerged, captured through the marked difference in the approach to training, support and leadership of women and families through their infant feeding journeys and beyond the original ‘Ten steps’ of the global BFHI standards.

#### 3.4.1 A step ahead—Transforming the performance

Staff, specifically the infant feeding leads, appeared to use transformational leadership qualities and approaches helping to move infant feeding care above and beyond the BFI standards. Their BFI work seemed to be ‘a feeding vocation—more than a job’ as captured by Julie:

Can I just say I think I have reached my dream now … I don't think it's still out there … I think this is it. This is my love, this is my life. (Julie, Infant Feeding Lead, Phase 1).

This was reflected by other leaders identified throughout the service, for example, one infant feeding support worker said: ‘You know, it's a passion of mine. So I absolutely adore what I do’ (IFSW, phase 3). This passion and commitment translated in to a sense of meaning—they felt their work mattered and had value.

Throughout all phases of data collection, it was clear that staff working closely with the BFI standards, especially the infant feeding leaders, were ‘going for GOLD with vision, beliefs and ideals’. Their vision and belief centred on the importance of breastfeeding and pushing beyond the boundaries of the BFI standards, as relayed by Lauren:

I think its winning hearts and minds really. I think, until you've got that bit, until you've got the belief then it's hard to put the actions in afterwards. Because the actions don't work without the belief and you'd have that as your kind of cornerstone. (Lauren, Feeding Champion and Volunteer, Phase 2).

To sustain this approach, the leaders were required to think ‘outside the box’ of the system, especially when resources were constrained:

But as time goes on and you develop leadership skills and you develop your experiences of working with baby friendly and you can start to think outside the box (Julie, infant feeding coordinator, Phase 1).

It was also important that the leaders and staff worked hard to get alongside each other and those in their care.

#### 3.4.2 Getting alongside part of the performance

The leaders prioritised 'being‐with' those they supported, rather than doing‐to them. For Julie, the infant feeding lead, it was clear that she wanted to participate rather than direct, enable, rather than perform. One of the midwives, working on the postnatal ward, discussed the value of the infant feeding leader being present, in relation to referring a breastfeeding mother and baby who needed support:

Straight away they were on the ball, there wasn't a delay and I didn't have to chase all the time it was just done’ (Shelley, midwife, Phase 1).

The leaders appeared to be staff‐friendly and approachable:

She's [the infant feeding lead] very approachable, she's always at the end of the phone, you know, she never makes you feels like she's—like you're troubling her, or anything like that. So I've been very lucky and I think that has minimised that feeling hugely, I think if it was a different manager I could have very easily felt a bit thrown into the deep end. (Judith, infant feeding midwife, Phase 2).

Being friendly to staff enabled a team approach to flourish. Working as a team seemed important to ensure the BFI standards were optimised throughout practice, I captured this in my reflective log:

Julie works with a range of feeding champions from across the hospital maternity setting and also the community. She is constantly looking for ways to recruit more volunteers and puts energy in to supporting these champions in practice through infant feeding champion meetings (Reflective log, Site 1).

Building leadership capacity was also an important part of developing a team approach:

So it's kind of building their confidence up, and their knowledge and their experience to know (Judith, Infant feeding midwife, Phase 3).

Modelling transformative leadership qualities appeared to enable effective sharing of knowledge and skills including the focus on mother, baby and family centred care.

#### 3.4.3 Coming full circle to mother, baby and family‐centredness

This basic theme captures how the revised BFI standards and various approaches of staff, particularly the infant feeding leaders, influenced attitudes and infant feeding care, coming full circle to mother, baby and family‐centredness. Throughout all phases of data collection, staff reported the overwhelmingly positive influence of the BFI on skin contact and latterly, with the revised standards, the promotion of close and loving relationships between all mothers, babies and their families. This was expressed by Kerry:

Instead of Aunty Doris saying ‘put your baby down—you're spoiling your baby’ don't keep picking them up for me that part of BFI, the responsiveness that's really important. That's what I discuss every day. Keep picking him up, keep giving him a kiss—make sure he is really secure and happy. That's what you want. It's [BFI] all special but that's a deal breaker for me. Because we've got the potential now to … because how many boys are there now that are jealous or insecure or got a bit of an attitude problem? You know, you've got the opportunity to change the world. (Kerry, Infant Feeding Support Worker, Phase 4).

Enhanced personalised conversations were observed more frequently following the introduction of the revised standards. This was reflected in both how the infant feeding leads centred training session around the individual needs of staff and, in turn, how staff used women‐centred and family‐focused conversations to explore infant feeding in practice.

So I mean the main thing, apart from the hour long visit what those midwives were doing is putting the mother central to the conversation and I kind of really train them not to dive into the checklist but first of all to find out where she's at right now, what she's thinking, what she's feeling, what her past experiences have been and building the information‐giving but have it as a communication, a conversation. (Julie infant feeding lead, Phase 1).

Staff reported the revised BFI standards being more holistic and more caring as captured by Judith:

Much more holistic. Much more caring. Much more, erm. Just open. Every woman can be involved in it [BFI revised standards] now in one way, shape or form. (Judith, infant feeding midwife, Phase 3)

Being with woman and baby, mother and baby friendly emerged more consistently following the introduction of the revised standards. I observed this in the interactions between women and staff, as reflected in my field notes:

The midwife said to the mother: ‘keep him close to you and try […] without any pressure or hurry, just to keep him close, and give him a chance to go to the breast.’ The mother appeared responsive, placing her baby in skin contact, remarking ‘haaah! It's lovely’. Then the midwife sat next to the woman as her baby attached at the breast for feeding (Field notes, Phase 3).

Women shared how these connections, between themselves and staff, influenced their experiences:

The staff were really friendly. They were really helpful. If I needed anything they were always there to support me … when I've looked at the other mums … they're just as well‐supported, so I'm 100% clear that it's not because I've just got a special baby that they're being nice to me. They're being nice to me because that's what they're like with everybody … they're being kind to everybody (Andrea, BF mother, Phase 3).

These experiences, shared by staff and families and captured during interactions observed, highlighted how the revised BFI standards encouraged a move beyond the original BFI ‘Ten steps’ with care that appeared both mother *and* baby friendly. These connections were further nurtured by key BFI leads, visible throughout the service, strengthened latterly with a ‘guardian’ reporting to hospital Trust board. These leaders were seen to motivate and maintain a focus on optimal infant feeding support, ‘coming full circle’ with the introduction of the revised BFI standards, back to woman‐, baby‐ and family‐focused care. These disseminated leaders appeared as essential components to the maternity unit's sustained success in maintaining the BFI standards, informing a more relational approach to infant feeding care provision as outlined in the revised BFI.

## 4 DISCUSSION

**FIGURE 2:**
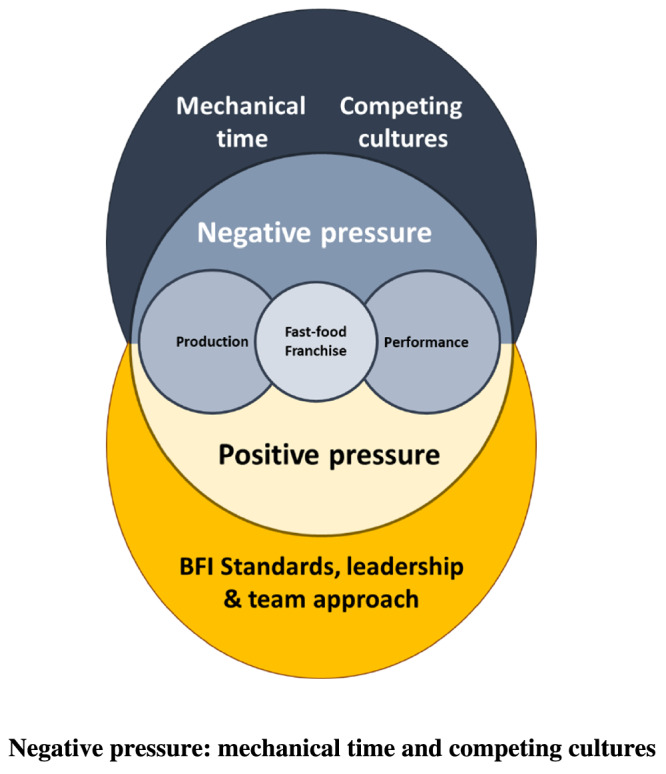
Converging and diverging pressures of providing, receiving and leading BFI in a fast food service

The findings presented in this paper demonstrate how the BFI standards were led, implemented and received within one maternity service in the UK over multiple years. The basic and organising themes, presented in the thematic network (Figure 1), capture how the BFI revised standards appeared to influence the experiences of working within and being cared for in a BFI accredited maternity unit. Our findings highlight how the BFI was provided and received by staff and service users, led by a team of motivated, passionate and committed infant feeding leaders who work to drive infant feeding care provision beyond the BFI, transforming infant feeding care. The findings highlight how these BFI providers, receivers and leaders face daily tensions between prioritising BFI and infant feeding and navigating the demands of the ‘fast‐food’ maternity hospital setting and service (captured in Figure 2).

### 4.1 Negative pressure: Mechanical time and competing cultures

Time constraints and the competing demands of the wider maternity service‐imposed threats to staff manageability, especially the midwives. The findings highlight how mechanical time informed the way care was organised and how the BFI was sustained from a moment‐to‐moment basis influenced by the pressures imposed on staff and service users. These findings resonate with existing knowledge regarding the influence of postnatal ward environments generally (Barker, 2013; Malouf, Henderson, & Alderdice, 2019; Sachs & Langlois, 2016; Wray, 2012) and in terms of infant feeding care provision (Dykes, 2005a, 2005b, 2006, 2009b; Hunter, Magill‐Cuerden, & McCourt, 2015; Schmeid, Beake, Sheehan, McCourt, & Dykes, 2011). Our findings outline the emotion work associated with sustaining BFI standards against the backdrop of the busy hospital maternity service. Findings that resonate with Furber and Thomson's (2008) research that outlined the ‘emotionalisation’ of infant feeding, identifying how midwives oscillate between positive and negative feelings in their practice depending on how much care they can offer. Midwifery emotion work has been well established in wider maternity care research (Deery & Hunter, 2010; Furber & Thomson, 2008; Hunter, 2001, 2002, 2004; Hunter & Deery, 2005; Morrow, McLachlan, Forster, Davey, & Newton, 2013). Our findings align with this work, outlining how the conflict and challenges midwives face between their ideals and the realities of their BFI and infant feeding support work have emotional consequences. However, the revised BFI standards, alongside the local leadership and team approach adopted, appeared as a resource to counter these pressures enabling midwives, and others offering infant feeding care, to generate a renewed sense of meaning and motivation in their work.

### 4.2 Positive pressure: BFI standards, leadership and team approach

Crucially, the revised BFI standards, combined with the local BFI leadership and team approach, converged to create a positive pressure sustaining the BFI production and implementation. The collective influence of these three factors enabled staff and service users to balance the tensions between the rational demands of the fast‐food maternity service setting, on the one‐hand, and optimal, relational infant feeding practices on the other. They appeared to offer staff and service users informational, practical and emotional support. This helped staff and service users to comprehend, manage and derive meaning from their infant feeding care and experiences informing the global theme: ‘Creating a BFI sense of coherence: providing, receiving and leading infant feeding care in a “fast‐food” maternity service’ as illustrated in Figure 1.

### 4.3 BFI sense of coherence

Sense of coherence (SOC) is a theoretical construct articulated by Antonovsky (1979, 1983). Coherence refers to a way of perceiving life experience that allows for the formation of adaptive human responses (Antonovsky, 1993a, 1993b). Antonovsky (1996) described SOC following a series of interviews that examined life histories and experiences. Crucially, he developed SOC as a way to explain how individuals transition between, what he described as, the health‐ease to dis‐ease continuum. Antonovsky (1996) proposed that the strength of an individual or collective SOC is shaped by three core life factors: life consistency, an underload‐overload balance and participation in meaningful decision making. From these factors, he established that SOC is generated from how comprehensible, manageable and meaningful life appears to be in any given moment (Antonovsky, 1985, 1987a, 1987b). By offering informational, practical and emotional support, the BFI standards, leadership and team approach observed at the study site created a BFI sense of coherence for staff and service users in terms of their infant feeding experiences. This was achieved by making infant feeding comprehensible (through information sharing), manageable (through practical support) and meaningful (by resonating emotionally). These insights support previous research that applied SOC theory in terms of how women want infant feeding support to be provided (Thomson & Dykes, 2011).

In terms of comprehensibility, BFI offered staff and service users a streamlined set of standards informing ‘the BFI way’. We found that the BFI seemed to encourage organised workflow and predictability in terms of infant feeding support. From this perspective, the BFI could be considered a health promoting resource for staff engaging with it. Our findings resonate with other studies that have shown that a predictable workflow context can increase comprehensibility and workplace well‐being in other contexts (Bringsen, Andersson, Ejlertsson, & Troein, 2012). Equally, the revised BFI appeared to create a culture where women and families seemed to be informed and supported with their feeding choices and breastfeeding were protected and facilitated, on the whole. These findings present as a contrast to previous research that has identified infant feeding support as inconsistent (Beake, Pellowe, Dykes, Schmied, & Bick, 2012; Ellberg, Hogberg, & Lindh, 2010; Schmeid, Beake, Sheehan, McCourt, & Dykes, 2011) and the general postnatal care and support offered as prescriptive (Fenwick, Butt, Dhaliwal, Hauck, & Schmeid, 2010). However, more recent research, by Groleau, Pizarro, Molino, Gray‐Donald, and Semenic (2016), aligns with the findings of this study, demonstrating how the BFI can enhance positive experiences and outcomes among mothers in Quebec, Canada. The infant feeding team appeared as the BFI glue—reinforcing the BFI standards throughout the service. They emerged as the key protectors, supporters and maintainers of the BFI and associated infant feeding practices.

The leaders appeared to use transformational leadership qualities to further enhance staff and service user comprehensibility. The infant feeding leads utilised what Bass (1999) refers to as ‘individualised consideration’ by paying attention to the developmental needs of other staff and service users. The leaders were seen to use individualised consideration, delegating BFI assignments and work, to stimulate shared understanding and opportunities for sustained BFI development throughout the workforce (Bass, 1999). BFI leads prioritised getting to know team members as individuals with personal goals and feelings. This seemed to allow the BFI leaders to provide staff with development opportunities and appeared to create a culture of caring among the team helping staff and service users to have ‘belief and motivation’ with regard to optimising infant feeding care. Balancing a rational, transactional approach to leadership with a relational approach appeared to reflect the paradigm shift of the revised BFI standards.

This move towards relationality appeared to align with midwifery‐centred philosophy and rhetoric. Staff, in this study, found the revised BFI more acceptable, creating less tension for implementing them into daily practice. Previous research has highlighted the dissonance and resistance competing paradigms can create in relation to breastfeeding support (Battersby, 2006, 2014; Leeming, Marshall, & Locke, 2017) or more generally throughout maternity care provision (Deery & Hunter, 2010; Hunter, 2004; Hunter & Deery, 2005; Kirkham, 2011). Our findings demonstrate that midwives and other maternity care staff moved beyond this resistance as they embraced the flexibility and autonomy that the revised BFI standards afforded them.

The service users appeared to value the balance of practical support and information with a relational and emotionally engaging approach from staff. Their care helped them to build confidence and feel reassured. These findings resonate with other research that has found support that is viewed by mothers as mother‐centred and responsive to their needs appears to be strongly valued, especially if it facilitates mothers' own decision making (Bäckström, Wahn, & Ekström, 2010; Hoddinott, Craig, Britten, & Mcinnes, 2012; Schmeid, Beake, Sheehan, McCourt, & Dykes, 2011).

The infant feeding team leaders generated enhanced meaning for staff and service users by utilising a mixture of top‐down (transactional) and bottom‐up (transformational) leadership styles, balancing rational and relational approaches to supporting staff and service users to work within and beyond the BFI standards. They adopted a hearts and minds approach, shown to be successful for BFI implementation in community settings of the UK (Thomson, Bilson, & Dykes, 2012). By addressing the hearts and minds of staff and service users the infant feeding leads, in our study, helped to enhance the manageability and meaningfulness of the BFI standards in practice. These findings echo the call for effective leadership and collaborative efforts with infant feeding care, identified as crucial for global BFHI implementation success with an increased focus on effective national leadership and coordination (Saadeh, 2012; UNICEF/WHO, 2017; WHO, 2017a).

Collectively, our findings outline three important factors for enabling SOC for staff and service users related to infant feeding practices and experiences: adopting the UNICEF UK BFI revised standards; effective infant feeding leadership and taking a team approach to infant feeding care. All three factors contributed to staff and service users feeling informed, supported and connected in terms of their infant feeding care provision and experiences.

#### 4.3.1 Strengths and limitations

This is the first ethnographic study to explore the influence the revised UNICEF UK BFI standards have on the organisational culture of one maternity unit. It has offered deeper qualitative insights into the consequences of these national BFI standards on staff and service users. Conducting the study over multiple years enabled observation and recording of changes in provider and receiver experiences following the implementation of the revised national UNICEF UK BFI standards. The focus of the research was on staff that work with the BFI standards every day, so was limited to midwives, infant feeding leads, students, peer supporters and maternity support workers. It would be useful to explore the experiences of neonatal staff—medics and nurses in future studies. Practical challenges or limitations are related to timing, eliciting trust, avoiding the ‘Hawthorne Effect’ and the researcher's subjectivity in interpretation. These were addressed, in part, through ‘ethnographic returning’, visiting the site over multiple phases and periods of time. It is impossible to entirely remove the impact of the Hawthorne effect, especially with the moderate level of participation observation employed. These observations offered important and interesting insights into the way staff implement and engage with BFI, alongside how service users experience BFI care provision and practices.

## 5 CONCLUSION

This critical ethnographic study focused on the cultural influences of the UNICEF UK BFI standards for both staff and service users in England. The findings from this research have direct relevance for maternity unit workers, infant feeding leaders, maternity units engaging in BFI implementation, the UNICEF UK BFI team and the international BFHI leads. More broadly, it offers health policy makers and managers of change valuable insight into how health interventions are implemented, adopted, maintained and embraced, in health settings, by staff and the influence they can have on service users. This research provides a resource for future investigations to draw from and expand upon the possible benefits and issues around large‐scale interventions to change practice and influence positive public health outcomes.

Ultimately, this research contributes to a greater sociological understanding of the BFI. Knowledge gained from this endeavour adds to an existing body of work understanding the cultural influence of the BFI on infant feeding care practices and experiences for staff and service users. It extends current theoretical conceptualisation of the discursive influences on practice and support. The findings from this study can subsequently inform breastfeeding policy, practice and the education of midwives and others working to support women, babies and families with their infant feeding care needs.

In conclusion, we argue that the BFI enables ‘informational’ (comprehensible), ‘practical’ (manageable) and ‘emotional’ (meaningful) support for both staff and service users. This is strengthened by effective, local leadership and a team approach. It is crucial that ongoing infant feeding policy, leadership and practice balance relational and rational approaches to generate positive infant feeding care provision and experiences.

## CONFLICTS OF INTEREST

The authors declare that they have no conflicts of interest.

## CONTRIBUTIONS

AB conducted the research. FD designed the research. FD, GT and MD supervised the research.
